# Raman Spectroscopy Enables Non-invasive and Confirmatory Diagnostics of Aluminum and Iron Toxicities in Rice

**DOI:** 10.3389/fpls.2022.754735

**Published:** 2022-05-16

**Authors:** Samantha Higgins, Sudip Biswas, Nicolas K. Goff, Endang M. Septiningsih, Dmitry Kurouski

**Affiliations:** ^1^Department of Biochemistry and Biophysics, Texas A&M University, College Station, TX, United States; ^2^Department of Soil and Crop Sciences, Texas A&M University, College Station, TX, United States; ^3^Institute for Quantum Science and Engineering, Texas A&M University, College Station, TX, United States

**Keywords:** metal toxicity, rice, Raman spectroscopy, non-invasive diagnostics, plants

## Abstract

Metal toxicities can be detrimental to a plant health, as well as to the health of animals and humans that consume such plants. Metal content of plants can be analyzed using colorimetric, atomic absorption- or mass spectroscopy-based methods. However, these techniques are destructive, costly and laborious. In the current study, we investigate the potential of Raman spectroscopy (RS), a modern spectroscopic technique, for detection and identification of metal toxicities in rice. We modeled medium and high levels of iron and aluminum toxicities in hydroponically grown plants. Spectroscopic analyses of their leaves showed that both iron and aluminum toxicities can be detected and identified with ∼100% accuracy as early as day 2 after the stress initiation. We also showed that diagnostics accuracy was very high not only on early, but also on middle (day 4–day 8) and late (day 10–day 14) stages of the stress development. Importantly this approach only requires an acquisition time of 1 s; it is non-invasive and non-destructive to plants. Our findings suggest that if implemented in farming, RS can enable pre-symptomatic detection and identification of metallic toxins that would lead to faster recovery of crops and prevent further damage.

## Highlights

- We show that Raman spectroscopy can be used for confirmatory pre-symptomatic diagnostics of high and medium levels of iron and aluminum toxicities in rice.

## Introduction

Continuous improvement in crop yield is critically important to address the growing problem of food security (Ingram and Maye, 2020; Payne and Kurouski, 2021). Biotic and abiotic stresses can substantially reduce crop yield. Biotic stresses caused by viruses, fungi, and bacteria can reduce up to 30% of the crop yield (Ingram and Maye, 2020; Payne and Kurouski, 2021), whereas abiotic stresses such as drought, flood, extreme temperatures, nutritional deficiencies, and metallic toxicities can be more detrimental (Mantri et al., 2012; Waqas et al., 2019; Angulo-Bejarano et al., 2021). These stresses can reduce up to 70% of the crop yield (Farber et al., 2019a; Lew et al., 2020; Payne and Kurouski, 2021). Some efforts have been made to subsequently reduce the impacts of various abiotic stresses in rice crops, through genetics, genomics, and breeding programs (Thomson et al., 2009; Chin et al., 2011; Jagadish et al., 2012; Hasanuzzaman et al., 2013; Kumar et al., 2014; Li et al., 2014; Matthus et al., 2015; Gonzaga et al., 2017; Singh et al., 2017; Septiningsih and Mackill, 2018; Thapa et al., 2020; Ignacio et al., 2021; Liang et al., 2021; Tnani et al., 2021).

Metal toxicities can damage the plant directly, as well as cause substantial health issues if such plants are consumed by animals and humans. For instance, aluminum (Al) toxicity is a major issue for crop production in numerous countries with acidic soils [soils with pHs of 5.5 and lower; (Barabasz et al., 2002)]. Such soils take over 50% of the arable land in the world. Al ions inhibit elongation and division of cells in the root tip, decelerating root development, which results in a substantial decrease in water and nutrient uptake by plants (Bojórquez-Quintal et al., 2017). This results in a substantial change in the plant metabolism and may cause the death of the plant (Farber et al., 2019a). Consumption of such Al-rich plants is also associated with dementia and other neurodegenerative disorders. Iron (Fe) is an essential element for all living organisms. However, if it accumulated in plants, Fe promotes Fenton reaction that generates hydroxyl radicals, which in turn damage lipids, proteins, and DNA (Sahrawat, 2005). Fe also inhibits cell division and elongation of plant roots. As a result, plants grown on Fe-rich soils demonstrate suppressed vegetation and lower crop yield compared to plants cultivated on soils with a low Fe content. These pieces of evidence demonstrate an urgent need for timely detection and identification of such metal toxicities in plants.

Conventionally, metal toxicities are diagnosed via colorimetric, atomic absorption or mass spectroscopy analyses (Sáez-Plaza et al., 2013). The colorimetric approaches are based on thiocyanates and a Prussian Blue dye (Woods and Mellon, 1941; Gitz et al., 2018). Such colorimetric approaches are routinely used for qualitative metal ion staining in animal and plant histological sections (Johnson et al., 2012; Roschzttardtz et al., 2013; Grillet et al., 2014; De la Fuente et al., 2017). However, these methods are laborious, lacks precision and fail to detect small variations of metals between samples. Atomic absorption spectroscopy is a highly accurate technique that is based on the electromagnetic emission of the individual elements that are converted into ions by flame-heating of the sample (Wachasunder and Nafade, 2001). Together with inductively coupled plasma-mass spectroscopy (ICP-MS), atomic absorption spectroscopy is frequently used for highly accurate identification of metal content of both plants and soils (Masson et al., 2010; Tokalıoğlu, 2013). However, these spectroscopy approaches are laborious and costly; they also require sample shipment to a laboratory, which typically cannot be afforded by most farmers in developing countries. These limitations of traditional techniques for plant and soil analyses catalyzed a search for inexpensive, fast, and portable analogs that can be used for confirmatory diagnosis of metal toxicities in plants.

Our own results, as well as experimental findings reported by other groups, show that Raman spectroscopy (RS) can be used to detect both biotic and abiotic stresses in plants (Altangerel et al., 2017; Egging et al., 2018; Farber and Kurouski, 2018; Farber et al., 2019b,^2021^; Mandrile et al., 2019; Sanchez et al., 2019b,2020a,b; Gupta et al., 2020). RS is based on a phenomenon of inelastic light scattering that was experimentally demonstrated by C.V. Raman at the beginning of the 20th century. These inelastically scattered photons provide information about the structure and composition of analyzed specimens (Kurouski et al., 2015). Biotic and abiotic stresses drastically alter plant metabolism (Payne and Kurouski, 2021). Such changes can be detected using RS, which allows for confirmatory, non-invasive, and non-destructive diagnostics of both biotic and abiotic stresses. For instance, Farber and Kurouski (2018) showed that RS could detect and identify different fungal pathogens in corn. Sanchez et al. (2019a,b, 2020c) discovered that RS could be used to detect Huanglongbing (HLB) or Citrus Greening Disease in oranges and grapefruits. Furthermore, using RS, HLB could be distinguished from nutritional deficiencies with 100% accuracy. Recently reported experimental findings show that RS can also detect and identify nutritional deficiencies caused by a lack of nitrogen, phosphorus, and potassium in soil (Sanchez et al., 2020a). These results show that RS be used to probe changes in plant biochemistry associated with a lack of macronutrients. Based on these findings, we hypothesized that RS may have similar sensitivity for diagnostics of changes associated with microelements, such as Al and Fe.

In this study, we aim to determine the extent to which RS can be used to detect Al and Fe toxicities in rice (*Oryza sativa*). For this, we performed a growth chamber experiment in which rice plants were exposed to medium and high levels of Al and Fe toxicities (Supplementary Figure 1). Using a hand-held Raman spectrometer, we collected spectra from leaves of these plants starting at day 2 (D2) after stresses were induced. These spectra were used to determine changes in plant biochemistry that are likely to be associated with both Al and Fe toxicities.

We also used Partial Least Squares-Discriminant Analysis (PLS-DA) to determine the accuracy of Raman-based diagnostics of metal toxicities. Our finding shows that RS coupled to PLS-DA can detect pre-systematic Al and Fe toxicities with high accuracies. These findings suggest that RS can be used for screening metal toxicities in plants. This information can be used to mitigate the stresses on the early stages of plant vegetation and minimize the consumption of such toxin-contaminated crops by animals and humans.

## Materials and Methods

### Plant Materials and Experimental Design

Presidio rice was grown in bins that contained Styrofoam with pads. The pads were covered by a mesh grid as described by Samonte et al. (2013) and Sanchez et al. (2020a). One pre-germinated seedling was placed in the mesh in each hole resulting in a total of 24 seedlings per bin. The seedlings were first soaked in water for the first 24 h to initiate seed germination (Sanchez et al., 2020a). Next, germinated plants were grown in a Yoshida solution consisting of macronutrients (114.30 mg/L NH_4_NO_3_, 50.40 mg/L NaH_2_PO_4_.2H_2_O, 89.30 mg/L K_2_SO_4_, 108.25 mg/L CaCl_2_ and 405 mg/L MgSO_4_.7H_2_O), and micronutrients [1.875 mg/L MnCl_2_.4H_2_O, 0.093 mg/L (NH_4_)_6_Mo_7_O_24_.4H_2_O, 1.09 mg/L H_3_BO_3_, 0.038 mg/L CuSO_4_.5H_2_O, 9.62 mg/L FeCl_3_.6H_2_O, 14.88 mg/L C_6_H_8_O_7_.H_2_O and 0.043 mg/L ZnSO_4_.7H_2_O] for 4 weeks. Next, plants were exposed to medium and high Fe and Al stresses. For Al stress, 0.042 mg/L or 314 μM of AlCl_3_ was used in the high-stress bin and 0.021 mg/L or 157 μM of AlCl_3_ was used in the medium stress bin. For Fe stress, the high-stress bin contained 2.196 g/L or 8.12 mM FeCl_3_.6H_2_O solution, and the medium stress bin contained 1.098 g/L or 4.06 mM FeCl_3_.6H_2_O solution. During the 2-week period in which plants were under metallic stress, height measurements, photographs, and Raman spectra were collected at D2, D4, D6, D8, D10, D12, and D14, Supplementary Figure 1. The pH was checked every day during stress conditions to ensure that the solution remained at a pH of 5. Every 3 days, a new hydroponic solution was created, the old stress solution was discarded, and fresh stress solutions were added at the same concentrations for each medium stress and high stress. The growth of the rice occurred in a controlled growth chamber with a day/night setting on a 12 h/12 h, humidity set to 55%, and a day/night temperature set to 29°C/26°C (Sanchez et al., 2020a). The experiment was repeated twice for this work.

### Raman Spectroscopy

Agilent Resolve hand-held Raman spectrometer was used to collect spectra from plants. For each spectral acquisition, the plant leaf was gently positioned at the nozzle of the spectrometer. Acquisition time was 1 s; laser power was 495 mW. Baseline correction was performed automatically by the spectrometer. These conditions were found to be non-destructive as no visual damage of plant leaves was evident after spectral acquisition (Sanchez et al., 2019a). A total of 50 spectra were collected from each group of plants (50 for control, 50 for Al high, 50 for Al medium, 50 for Fe high, and 50 for Fe medium). Next, spectra were exported from the spectrometer and analyzed using PLS Toolbox (Eigenvector Research Inc., Wenatchee, WA, United States).

### Data Analysis

First, multiplicative signal correction based on the mean was applied to all data. Next, the second derivative was taken of the Raman spectra with a filter width of 51 and polynomial order 3. Finally, the spectra were smoothed with a 15-point window then area normalized. A partial least squares-discriminant analysis (PLS-DA) was performed for all data presented in the results and discussion of this manuscript. The imported spectra wavenumbers that were analyzed were included from 300 cm^–1^ to 2,000 cm^–1^ which includes all important spectra characteristics of rice plants and metallic stress. Each spectrum was area normalized at 1,440 cm^–1^ and mean-centered. Every PLS model statistically analyzed the level of metallic stress to sort the significant differences between Al and Fe metallic toxicities, Supplementary Figure 2. PLS-DA results are summarized in Tables 2, 3. For instance, Al high stress plants were compared to the control group (healthy plants) on D2 using the PLS-DA binary model in which the Cross Validation matrix ranked the accuracy of the ability for the Raman spectrometer to distinguish between the two data sets. The confusion matrix of Al high versus healthy on D2 gave a true prediction rate (TPR) of 1 (100%) for distinguishing Al High stress plants from healthy plants. The same method was used for the other experimental groups and following days (see Supplementary Figures 1, 2).

## Results and Discussion

**FIGURE 1:**
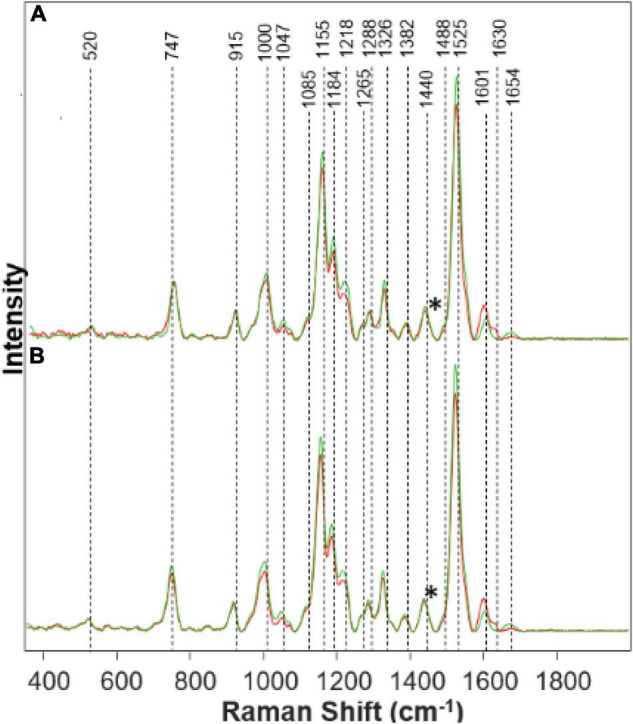
Raman spectra collected from leaves of healthy (green) and Fe stressed (red) at day 10 with high **(A)** and medium **(B)** levels of Fe. Spectra normalized on CH_2_ vibrations (1,440 cm-1) present in nearly all classes in biological molecules [marked by asterisks (*)].

Spectra collected from leaves of healthy rice plants exhibited vibrational bands that could be assigned to pectin (747 cm^–1^), cellulose (915 and 1,048 cm^–1^), carotenoids (1,000, 1,155, 1,185, 1,218, and 1,525 cm^–1^), phenylpropanoids (1,601–1,630 cm^–1^), protein (1,674 cm^–1^), and aliphatic vibrations (1,218, 1,288, 1,326, 1,382, 1,440, and 1,488 cm^–1^) that cannot be assigned to a particular class of compounds (Figure 1 and Table 1).

**TABLE 1:** Vibrational bands and their assignments for spectra collected from rice leaves.

Band (cm^–1^)	Vibrational mode	Assignment
520	ν(C-O-C) Glycosidic	Carbohydrates (Edwards et al., 1997; Pan et al., 2017)
747	γ(C–O-H) of COOH	Pectin (Synytsya et al., 2003)
915	ν(C-O-C) In-plane, symmetric	Cellulose, phenylpropanoids (Edwards et al., 1997)
1,000	In-plane CH_3_ rocking of polyene aromatic ring of phenylalanine	Carotenoids (Schulz et al., 2005); protein
1,047	ν(C-O)+ν(C-C)+δ(C-O-H)	Cellulose, phenylpropanoids (Edwards et al., 1997)
1,085	ν(C-O)+ν(C-C)+δ(C-O-H)	Carbohydrates (Almeida et al., 2010)
1,155	C-C Stretching; ν(C-O-C), ν(C-C) in glycosidic linkages, asymmetric ring breathing	Carotenoids (Schulz et al., 2005), carbohydrates (Wiercigroch et al., 2017)
1,184	ν(C-O-H) Next to aromatic ring+σ(CH)	Carotenoids (Schulz et al., 2005)
1,218	δ(C-C-H)	Carotenoids (Schulz et al., 2005), xylan (Agarwal, 2014)
1,265	Guaiacyl ring breathing, C-O stretching (aromatic); -C=C-	Phenylpropanoids (Cao et al., 2006), unsaturated fatty acids (Jamieson et al., 2018)
1,288	δ(C-C-H)	Aliphatics (Yu et al., 2007)
1,326	δCH_2_ Bending	Aliphatics, cellulose, phenylpropanoids (Edwards et al., 1997)
1,382	δCH_2_ Bending	Aliphatics (Yu et al., 2007)
1,440–1,488	δ(CH_2_)	Aliphatics (Yu et al., 2007)
1,525	-C=C- (in-plane)	Carotenoids (Rys et al., 2014; Adar, 2017; Devitt et al., 2018)
1,601–1,630	ν(C-C) Aromatic ring+σ(CH)	Phenylpropanoids (Agarwal, 2006; Kang et al., 2016)
1,654	-C= C-, C=O Stretching, amide I	Proteins (Devitt et al., 2018)

We found that spectra collected from leaves of rice exposed to both medium and high Fe toxicities demonstrate an increase in the intensities of phenylpropanoid vibrations (1,601–1,630 cm^–1^), as well as a decrease in the intensities of carotenoids (1,000, 1,155, 1,184, 1,218, and 1,525 cm^–1^). These spectral changes suggest that Fe toxicity is associated with an increase in the concentration of phenylpropanoids and a decrease in the concentration of carotenoids.

Our own results, as well as experimental findings reported by other groups, show that on the molecular level, an increase in the intensity of phenylpropanoid vibrations is due to an increase in the concentration of low molecular weight aromatic compounds, such as p-coumaric acid. These molecules are also directly involved in the plant response against biotic and abiotic stresses such as bacteria and nutritional deficiencies.

A decrease in the carotenoid content has strong physiological relevance to plant defense mechanisms (Havaux, 2014). Specifically, Fe ions activate enzymatic oxidation of neoxanthin, one of the plant carotenoids, that yields abscisic acid, a hormone that enhances plant resistance to such stresses (Nambara and Marion-Poll, 2005). Reactive oxygen species (ROS) produced by Fenton reaction can also oxidize β-carotenes producing β-lonone, β-cyclocitral that aim to protect the plant against various biotic and abiotic stresses (Nambara and Marion-Poll, 2005; Havaux, 2014). Thus, reduction in the concentration of carotenoids by ROS can be considered to be a hallmark of Fe toxicity (Yu et al., 2007).

We have also observed a decrease in the intensity of cellulose (1,047 cm^–1^) and protein (1,654 cm^–1^) bands (Figure 1). These spectral changes that Fe toxicities are associated with cellulose degradation in plants, as well as the strong transformation of plant enzymes. More detailed elucidation of direct biochemical changes requires the use of high-performance liquid chromatography (HPLC) and its mass spectroscopy (HPLC-MS) analogs. These studies are beyond the scope of the current work.

The physiological effects of Fe on plant health corresponds with what is shown on the Raman spectra in Figure 1. The increased peak in the phenylpropanoid band region corresponds to an increase in phenylpropanoids within the plant. Phenylpropanoids play a key role in plant development and cell division. Thus, when metal toxicity such as Fe is present, there is an increase in phenylpropanoids to defend itself against abiotic stresses (Cheynier et al., 2013). These phenolics can be detrimental to the plant when released as a defense mechanism due to the toxicity of some phenols in great quantities, which causes harm to plant growth (Cheynier et al., 2013). Visual evidence is seen in the Supplementary Material of Fe stressed plants that they died the quickest. Carotenoids are partly responsible for plant color and photosynthesis. We see that the Fe stress plants quickly lost their color, turning brown rapidly. A decrease in the carotenoid band region corresponds to a decrease in carotenoids in the Fe stressed plants, leading to a lack of healthy color. Without carotenoids, the plant will lose molecules for maintaining normal plant health. Carotenoids are a predecessor to abscisic acid (ABA) which is involved in a defense mechanism (Stanley and Yuan, 2019). Since carotenoids play a role in the photosynthetic process, it is evident that with a decrease in carotenoids, the plant will become extremely sick or die (as we see in both Fe medium stress and Fe high-stress plants). Proteins and pectin are integral components for the strength and structure of the plant. Pectin is responsible for cell wall strength (Wu et al., 2018). Moreover, we see a decrease in the protein and pectin band intensities corresponding to the reduction in pectin and protein concentration within the Fe stressed plants also leading to degraded plants shown visually from the Supplementary Material.

**FIGURE 2:**
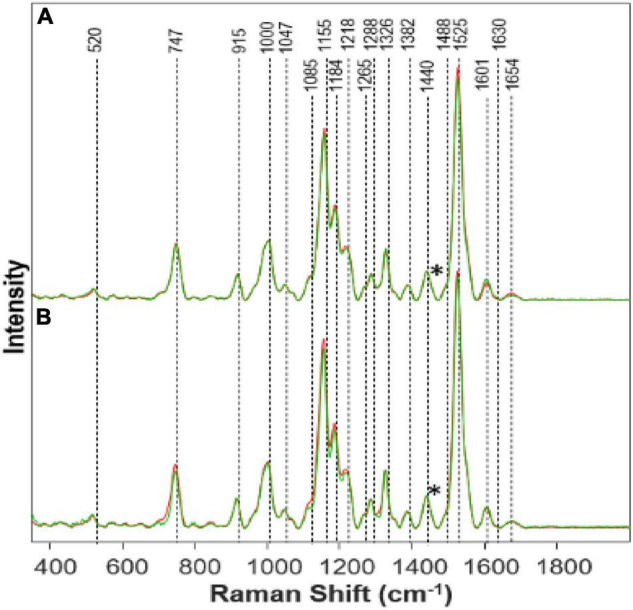
Raman spectra collected from leaves of **(A)** Al High (red) vs. Healthy (green) on day 14; **(B)** Al Medium (red) vs. Healthy (green) on day 14. Spectra normalized on CH_2_ vibrations (1,440 cm-1) present in nearly all classes in biological molecules [marked by asterisks (*)].

We found that Al toxicities cause nearly opposite changes in plant biochemistry. Specifically, we have observed an increase in the intensities of carotenoid and protein vibrations in the spectra collected from plants exposed to Al stresses (Figure 2). We also found that the intensity of phenylpropanoid vibrations decreased in the spectra collected from Al-stressed plants. Importantly, if the magnitude of these changes for medium and high Fe stresses were nearly the same, the magnitude of stresses for high (Figure 2A) vs. medium (Figure 2B) Al stresses is different. As expected, we have observed greater changes upon stronger (high vs. medium) stresses.

Visually the Al stressed plants appeared healthier and grew taller while keeping the healthy green color for a longer time. Much is still to be learned from Al stress and why an opposite effect was shown comparatively with Fe stress. This is quite interesting because the phenylpropanoid band intensity decreases as a representation of the decrease in phenylpropanoids under Al stress. This, in turn, should mean that the root elongation would also decrease rapidly, and the Al stress plants would die quickly. However, we see that the Al high-stress plants grew the most in comparison to the other stressed plants. It is possible that Presidio may possess some degree of tolerance to Al toxicity. It is known that Al will affect different varieties of plants in different ways (Bojórquez-Quintal et al., 2017). Therefore, further research needs to be done especially on the physiological effects of Al toxicity in plants.

Other studies have shown that Al can induce a more efficient nutrient uptake mechanism which would lead to faster growth (Bojórquez-Quintal et al., 2017). This may explain the reason we see rapid growth yet a decrease in the phenylpropanoid band because the efficiency of nutrient uptake is combatting the decrease in phenylpropanoids. The pectin band increased while the concentration of Al increased, suggesting an increase of pectin within the plant. Another study also found an increase of pectin by 50% in Al stress plants (Nagayama et al., 2019). Pectin is responsible for cell wall strength (Wu et al., 2018). Pectin can be linked to increased plant growth (Hassan et al., 2019). Due to Al influencing pectin increase within the plant, and pectin aiding in growth, we see an increase in the height of the Al high-stress plants. With the increase in the carotenoid band intensity, as Al concentration increases, an inference can be made that Al has caused an increase in the concentration of carotenoids within the plant. Carotenoids are linked to plant growth and are also produced as part of a defense mechanism when certain biotic and abiotic stresses are present (Swapnil et al., 2021). Since carotenoids are an integral part of photosynthesis, it can be stated that the increase in Al causing an increase in carotenoids has led to faster growth. Higher carotenoid concentration will allow for more efficient photosynthesis and thus plant growth. We also see that the protein band has an increased intensity, meaning an increased concentration in protein within Al stress plants. Previous studies have also found that Al can increase a variety of proteins within a plant (Chen and Lin, 2010). Proteins also aid in plant growth, as we see in the Al high-stress plants. Again, further study should investigate the effects of Al on plant growth and toxicity.

Next, we used PLS-DA to investigate the accuracy of identifying both Al and Fe stresses at different states of plant vegetation (Table 2). We found that both Al and Fe stresses (high and medium levels) can be diagnosed as accurately as 98% at D2. This accuracy slightly decreases at D4 (83%), then reaching 100% at D6. These findings suggest that once the stress is induced, it causes drastic changes in plant biochemistry (D2). However, aiming to mitigate such stresses, plants try to level off Al- and Fe- induced changes in plant biochemistry, which reduces the accuracy of their diagnostics at D4. The magnitude of such efforts is reduced at D6, which results in highly accurate differentiation between the biochemistry of a healthy plant and plants exposed to both Fe and Al toxicities.

**TABLE 2:** Percent accuracy of stress vs. healthy over 2 weeks.

Accuracy of stress vs. Healthy each day							

	D2	D4	D6	D8	D10	D12	D14
Al High	100%	82%	100%	92%	94%	90%	96%
Al Medium	100%	94%	100%	100%	74%	100%	94%
Fe High	92%	82%	100%	100%	100%	–	–
Fe Medium	100%	74%	100%	100%	100%	100%	92%

**FIGURE 3:**
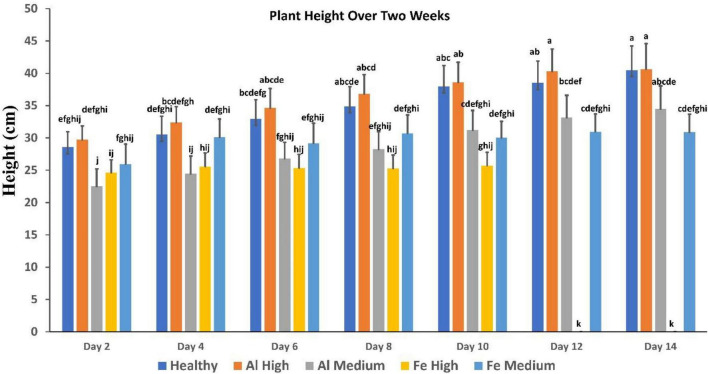
Comparison of plant height over 2 weeks, showing healthy vs. stress. Fe died after day 10. Each bar represents the mean ± SE (*n* = 14). Different letters in each graph (a–k) indicate significant differences (*P* < 0.05, ANOVA and Duncan’s test).

We also investigated the extent to which spectroscopic libraries can be used for robust and reliable identification of these stresses in plants. For this, we used spectra collected at different time points to build a model that can be used for the identification of high and medium Al and Fe stresses (Figure 3). We found that high Al stress can be diagnosed with 88.6% accuracy, whereas high Fe stress can be detected and identified with 88.3% accuracy (Table 3). Interestingly, the accuracy of diagnostics of medium levels of stresses is higher and is equal to 94.9% and 88%, respectively. Finally, we found that the absence of metal stresses healthy plants can be diagnosed with ∼90% accuracy.

**TABLE 3:** Percent accuracy from binary model to distinguish between stressed plant and healthy plants.

Stress vs. Healthy			

	Al	Fe	Healthy Avg.
High stress	88.60%	87.23%	88.29%
Medium stress	94.90%	88.03%	91.00%

It is important to note that conventionally used parameters, such as plant height, cannot be used for the identification of such changes (Figure 3). Specifically, we found that plants exposed to high Al stresses are taller than healthy plants, whereas analogous medium Al stresses result in substantial deceleration of the plant growth. High Fe stresses cause substantial impairment to the plant vegetation that results in substantial deceleration of the plant growth. However, plants exposed to medium Fe stress, although they experience some impairment in the plant growth typically only incrementally behind healthy plants at the corresponding vegetation state.

It is important to note that this work is a first proof-of-principle study that reflects the potential of RS in confirmatory diagnostics of plant stresses caused by metal toxicities. Additional work is required to verify these findings on other crops, such as sorghum, wheat and corn. It is also important to investigate the extent to which a spectroscopic library developed for one plant variety in one geographic area can be used for the accurate stress determination in other varieties and other geographic areas. Finally, it is critically important to determine the extent to which other abiotic stresses, as well as biotic stresses that can be simultaneously present in plants that experience metallic toxicities can alter the accuracy of detection of individual stresses. These studies are currently in progress in our laboratory.

## Conclusion

Our findings show that RS can be used for a label-free, fast and confirmatory diagnostics of plant stresses caused by Al and Fe toxicities. Although such diagnostics can be performed by atomic absorption spectroscopy and ICP-MS, RS requires no sample modification which drastically limits direct costs of such testing. Our results show that already at D2, one can detect and identify medium when referring to the stress levels and high Al and Fe toxicities with ∼100% accuracy. Our findings demonstrate that not only at early but also at middle and late stages, both middle and high Al and Fe toxicities can be correctly detected and identified. One can expect that a hand-held Raman spectrometer equipped with such libraries and chemometric models can be used directly in the field for timely assessment of the plant health. We also expect that such devices can be used in grocery stores for label-free, non-invasive and non-destructive control of plant products.

This work also expanded the potential of RS in digital farming. Our findings together with experimental results reported by other groups demonstrate that this innovative spectroscopic technique can be used to transform agricultural approaches in the United States and overseas (Altangerel et al., 2017; Egging et al., 2018; Farber and Kurouski, 2018; Farber et al., 2019b,^2021^; Mandrile et al., 2019; Sanchez et al., 2019b,2020a,b; Gupta et al., 2020).

## Data Availability Statement

The original contributions presented in the study are included in the article/Supplementary Material, further inquiries can be directed to the corresponding author/s.

## Author Contributions

SH: investigation, data curation, and methodology. SB: methodology. NG: investigation. ES: methodology and supervision. DK: methodology, funding acquisition, and supervision. All authors contributed to the article and approved the submitted version.

## Conflict of Interest

The authors declare that the research was conducted in the absence of any commercial or financial relationships that could be construed as a potential conflict of interest.

## Publisher’s Note

All claims expressed in this article are solely those of the authors and do not necessarily represent those of their affiliated organizations, or those of the publisher, the editors and the reviewers. Any product that may be evaluated in this article, or claim that may be made by its manufacturer, is not guaranteed or endorsed by the publisher.
