# Supplementary material for: Raman Spectroscopy Enables Non-invasive and Confirmatory Diagnostics of Aluminum and Iron Toxicities in Rice
Source: Front Plant Sci. 2022 May 16;13:754735. doi: 10.3389/fpls.2022.754735 (PMC9149412; doi:10.3389/fpls.2022.754735)
Supplement: Supplementary file 1 [file Table_1.pdf]

Raman Spectroscopy Enables Non-Invasive and Confirmatory Diagnostics of Aluminum and Iron Toxicities in Rice

Samantha Higgins[1], Sudip Biswas[2], Nicolas K. Goff[1], Endang M. Septiningsih[2], and Dmitry Kurouski[*1,3]

1. Department of Biochemistry and Biophysics, Texas A&M University, College Station, Texas 77843, United States
2. Department of Soil and Crop Sciences, Texas A&M University, College Station, Texas 77843, United States
3. The Institute for Quantum Science and Engineering, Texas A&M University, College Station, Texas, 77843, United States

Highlights: We show that Raman spectroscopy can be used for confirmatory pre-symptomatic diagnostics of high and medium levels of iron and aluminum toxicities in rice.

Supporting Information

*****Note: Nitrogen High and Medium images are excluded for the relevance of the supplementary material.

Photographs

Day 2

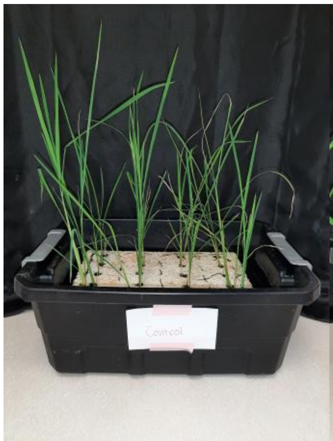

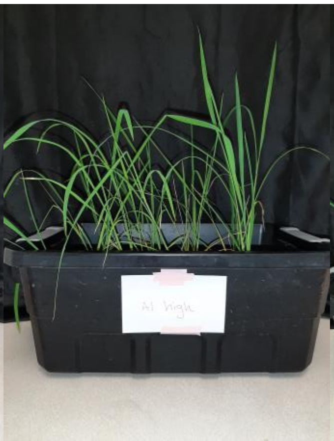

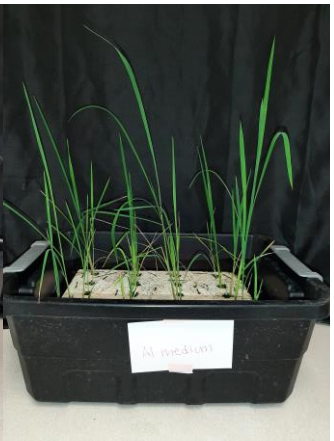

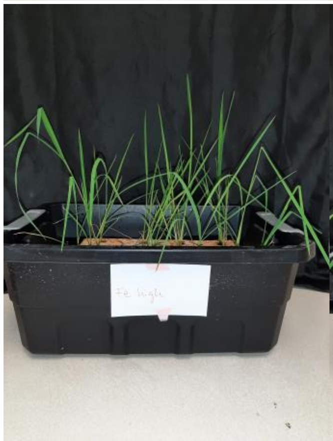

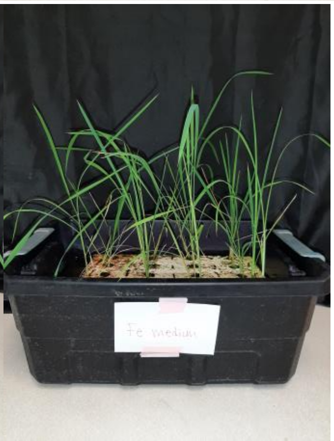

Day 4

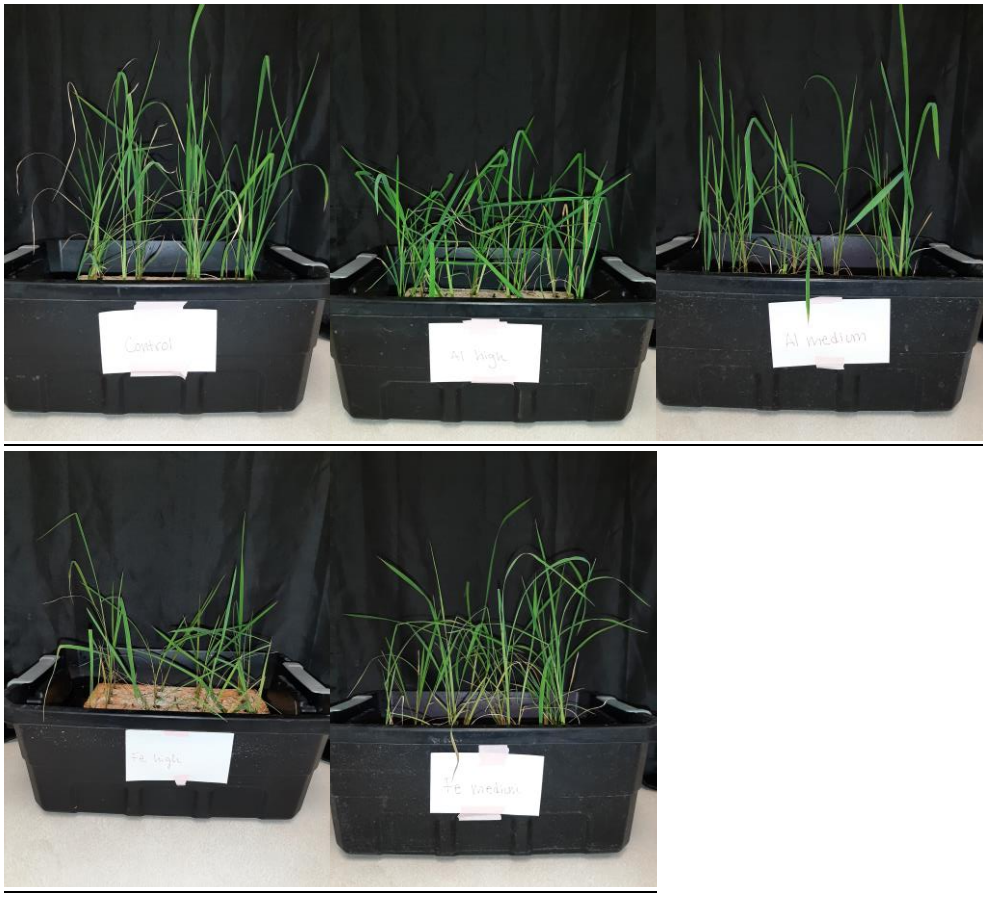

<u>Day 6</u>

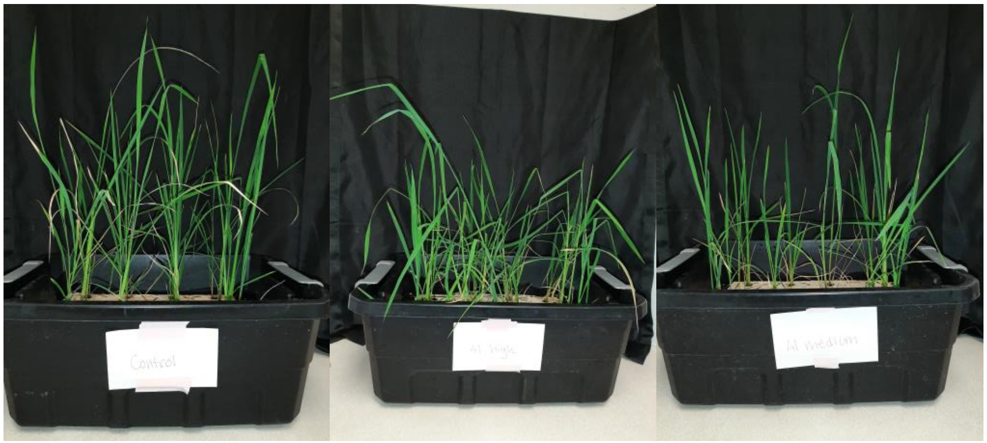

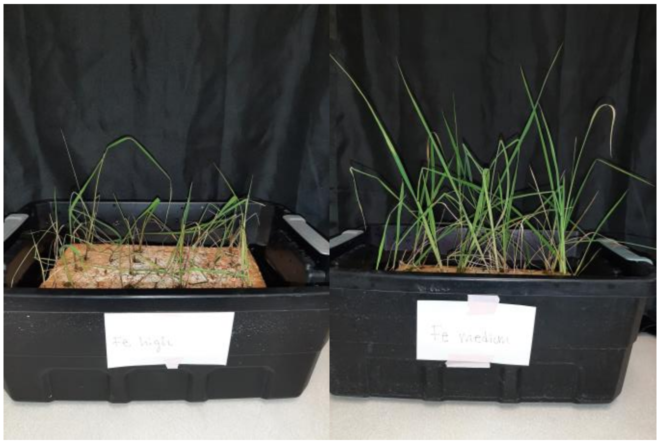

Day 8

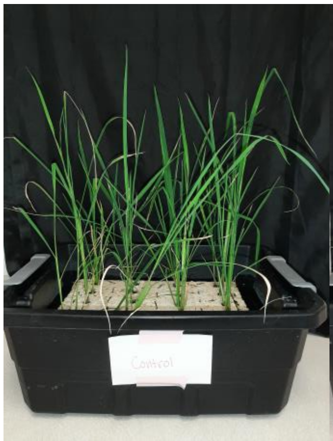

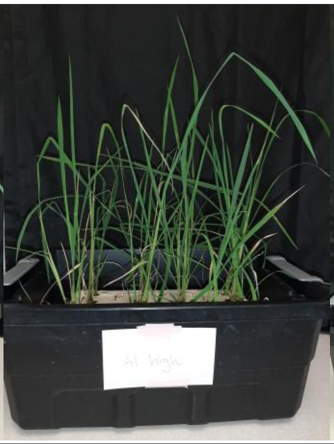

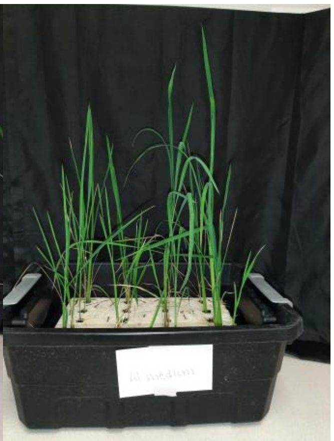

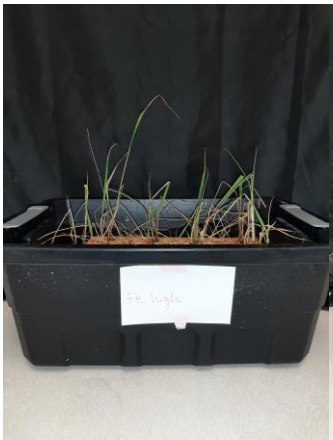

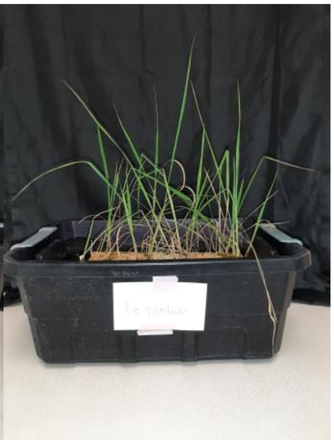

Day 10

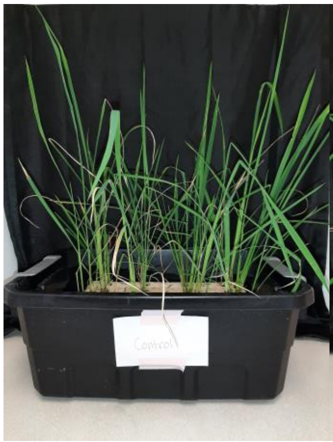

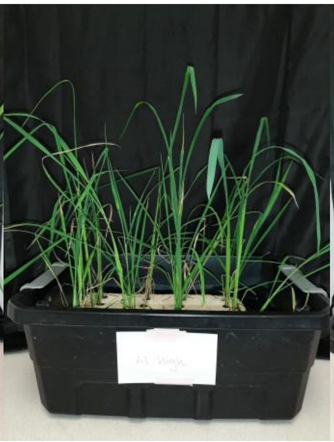

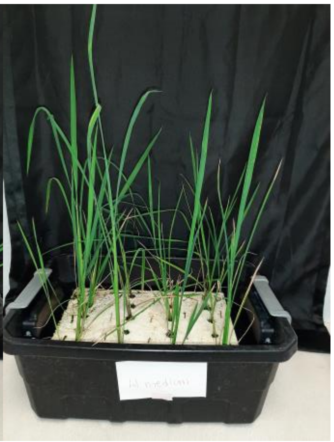

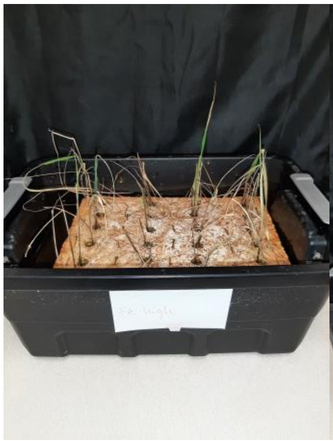

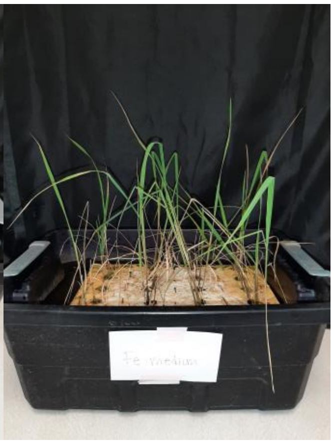

Day 12

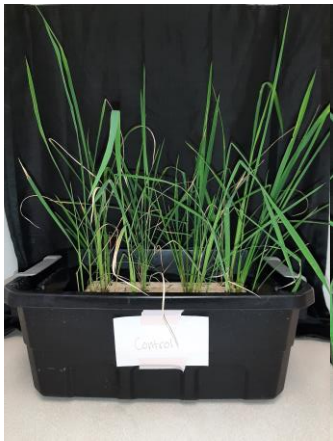

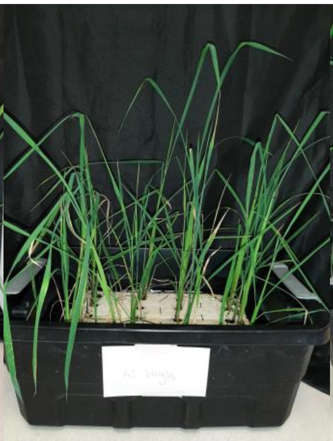

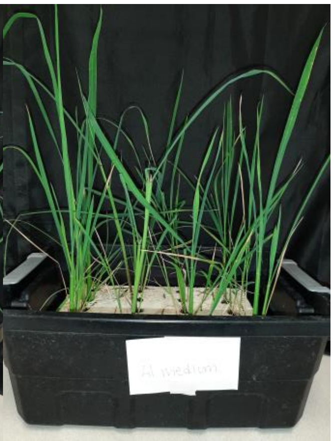

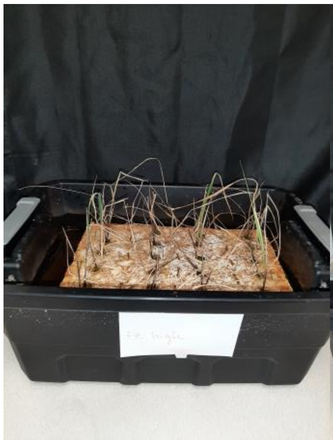

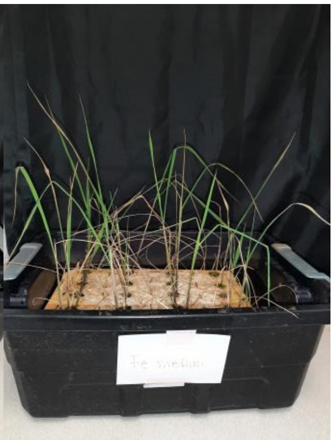

Day 14

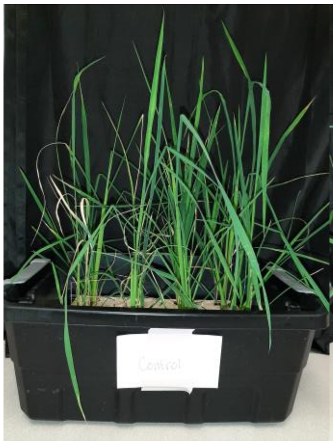

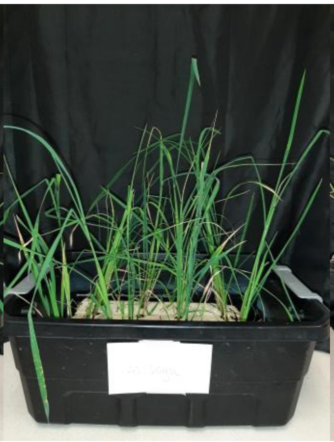

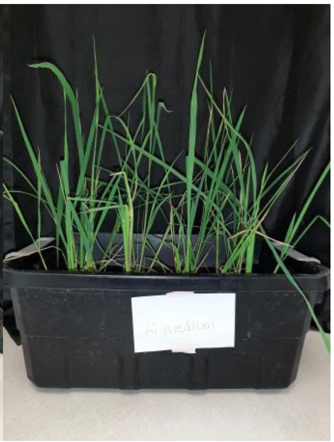

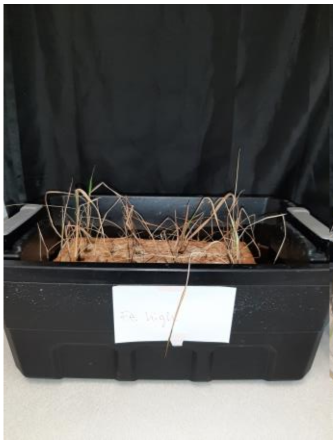

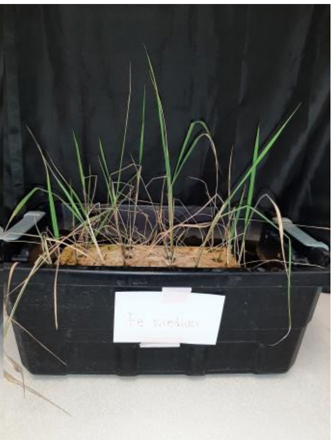

Figures

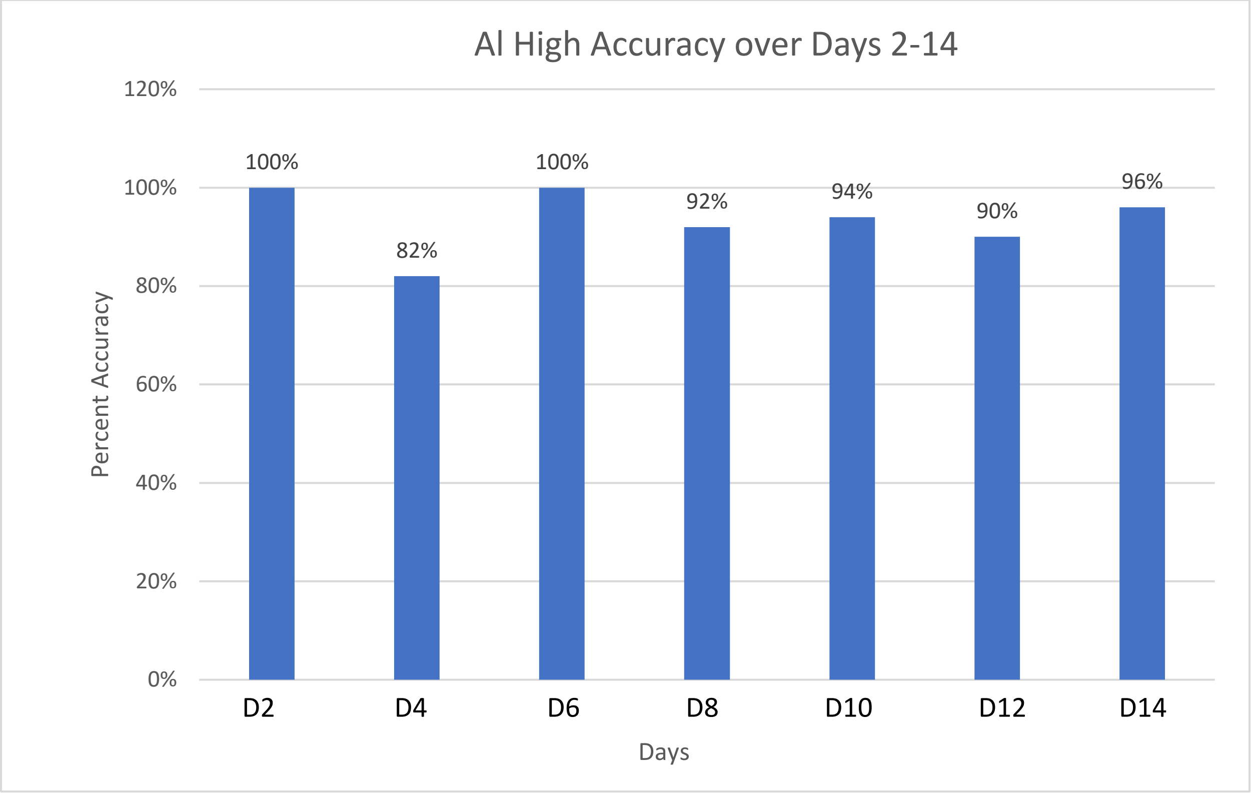
AI High Accuracy over Days 2-14
120%
100%
80%
60%
40%
20%
0%
Percent Accuracy
100%
82%
100%
92%
94%
90%
96%
D2
D4
D6
D8
D10
D12
D14
Days

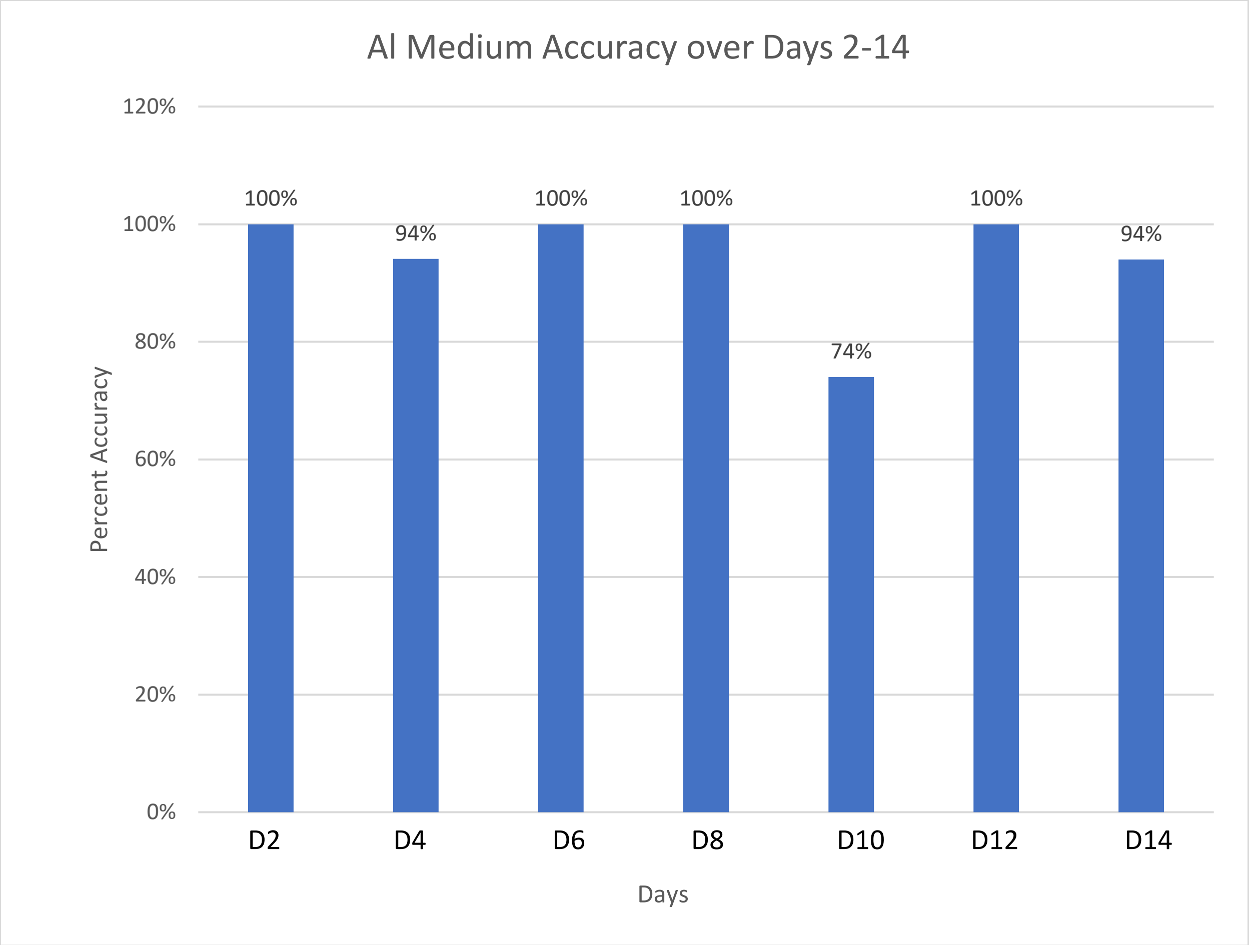
Al Medium Accuracy over Days 2-14
Percent Accuracy
120%
100%
80%
60%
40%
20%
0%
100%
94%
100%
100%
74%
100%
94%
D2
D4
D6
D8
D10
D12
D14
Days

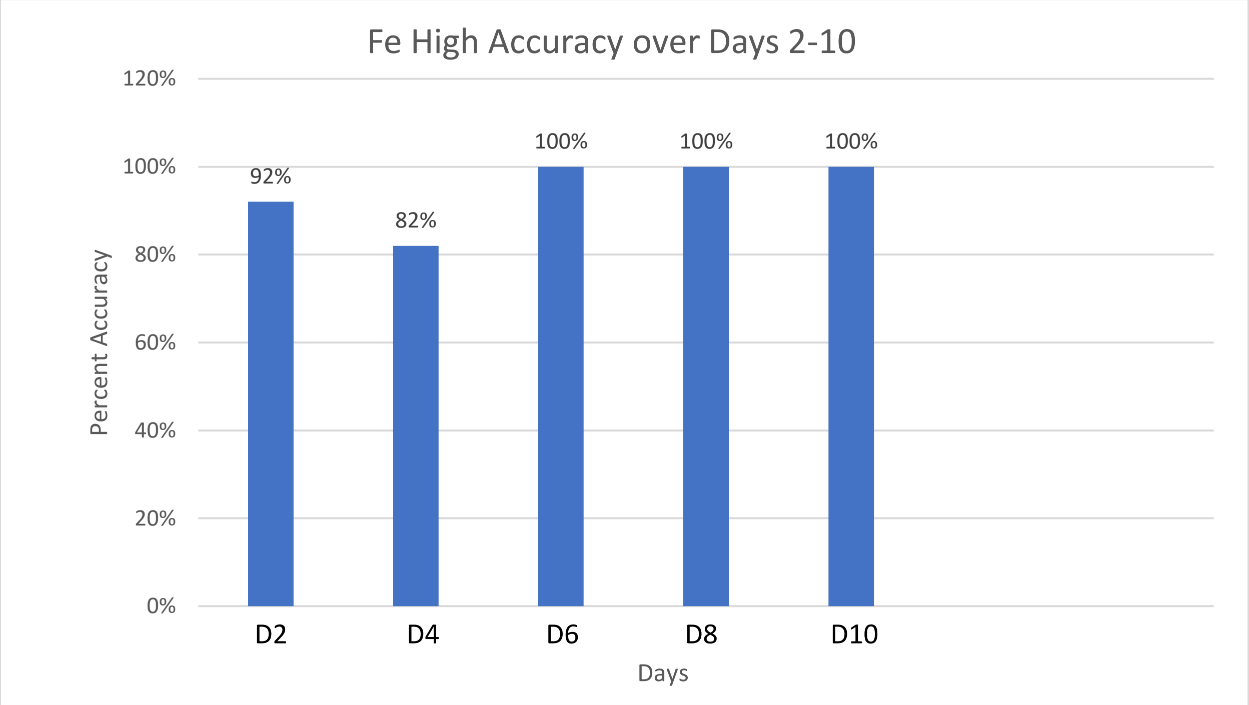
Fe High Accuracy over Days 2-10
Percent Accuracy
120%
100%
80%
60%
40%
20%
0%
92%
82%
100%
100%
100%
D2
D4
D6
D8
D10
Days

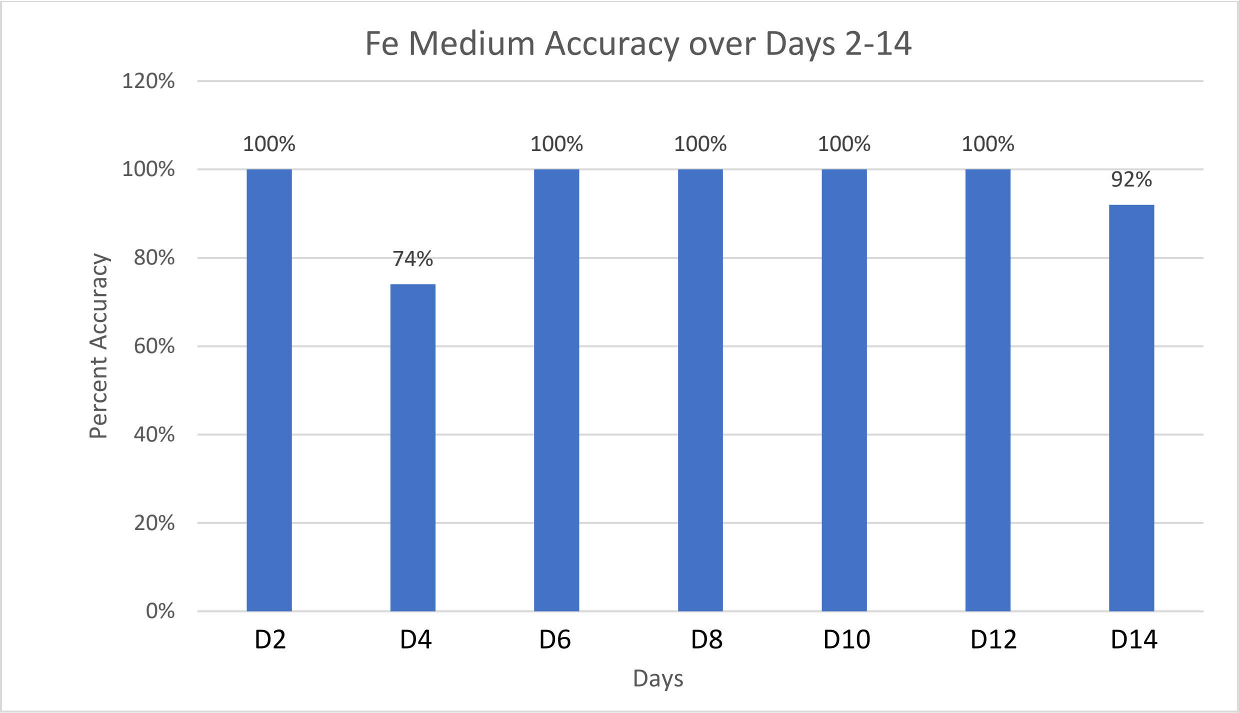
Fe Medium Accuracy over Days 2-14
120%
100%
80%
60%
40%
20%
0%
Percent Accuracy
100%
74%
100%
100%
100%
100%
92%
D2
D4
D6
D8
D10
D12
D14
Days
